# Effect of *GAS6* and *AXL* Gene Polymorphisms on Adiposity, Systemic Inflammation, and Insulin Resistance in Adolescents

**DOI:** 10.1155/2014/674069

**Published:** 2014-02-18

**Authors:** Fone-Ching Hsiao, Yuh-Feng Lin, Po-Shiuan Hsieh, Nain-Feng Chu, Yii-Der Ida Chen, Yi-Shing Shieh, Chang-Hsun Hsieh, Chien-Hsing Lee, Ting-I Lee, Yi-Jen Hung

**Affiliations:** ^1^Graduate Institute of Clinical Medicine, College of Medicine, Taipei Medical University, Taipei City 110, Taiwan; ^2^Division of Endocrinology and Metabolism, Department of Internal Medicine, Tri-Service General Hospital, National Defense Medical Center, Taipei City 114, Taiwan; ^3^Department of Physiology and Biophysics, National Defense Medical Center, Taipei City 114, Taiwan; ^4^School of Public Health Department, National Defense Medical Center, Taipei City 114, Taiwan; ^5^Los Angeles Biomedical Research Institute, Harbor-UCLA Medical Center, Torrance, CA 90509, USA; ^6^Department of Oral Diagnosis and Pathology, School of Dentistry, Tri-Service General Hospital, National Defense Medical Center, Taipei City 114, Taiwan; ^7^Division of Endocrinology and Metabolism, Wan Fang Hospital, Taipei Medical University, Taipei City 116, Taiwan

## Abstract

The present study was designed to explore the effects of *GAS6 *and *AXL* gene polymorphisms on adiposity, systemic inflammation, and insulin resistance in adolescents. After multistage sampling from the data of the Taipei Children Heart Study-III, we collected 358 boys and 369 girls with an average age of 13.3 years. We genotyped the adolescents' *GAS6* rs8191973, *GAS6* rs8191974, *AXL *rs4802113, and *AXL* rs2304232 polymorphisms. Significantly higher body mass index (BMI), waist circumference (WC), and hsCRP levels were found in boys with the GG genotype of *GAS6* rs8191974 than A allele carriers; higher IL-6 and insulin levels and increased HOMA-IR were found in boys with the GG genotype of *AXL* rs2304232 than the A allele carriers. There was a significant difference in hsCRP levels of boys with the TT, TC, and CC genotypes of *AXL* rs4802113. Boys with both the GG genotype of *GAS6* rs8191973 and the GG genotype of *GAS6* rs8191974 exhibited higher BMI, WC, IL-6, and hsCRP levels than the boys carrying both the C allele of the *GAS6* rs8191973 and the A allele of the *GAS6* rs8191974. In conclusion, *GAS6* and *AXL* polymorphisms are associated with adiposity, systemic inflammation, and insulin resistance in adolescents, especially in boys.

## 1. Introduction 

Childhood obesity is a serious and growing public health problem that has arisen over the past three decades [1]. The increasing occurrence of disorders such as type 2 diabetes during childhood is believed to be a consequence of this obesity epidemic [1]. In addition to several behavioral and dietary risk factors, genetic predisposition is an important factor in the pathogenesis of childhood obesity [2]. It is estimated that 40–70% of adiposity variance can be explained by direct or indirect genetic factors [3].

Growth arrest-specific 6 (Gas6), cloned in 1988 and characterized in 1993, is a secreted vitamin K-dependent protein present in the human circulatory system [4, 5]. Initially, Gas6 was shown to be upregulated in growth-arrested fibroblasts, suggesting that it plays a protective role in certain cellular stresses such as during apoptosis [6]. Gas6 expression is widespread in many tissues, including immune cells, endothelial cells, vascular smooth muscle cells, and adipocytes [7–9]. The protein is also a ligand for the TAM (Tyro-3/Axl/Mer) family tyrosine kinase receptor [4]. The Gas6/TAM system has been implicated in cell survival and proliferation, cell adhesion and migration, hemostasis, and inflammatory cytokine release [4, 10].

Recently, the Gas6/TAM pathway was found to be involved in mediating adipocyte survival and proliferation *in vitro *[11, 12]. Experiments with mice fed a high-fat diet indicated that overexpression of Gas6 might enhance body-fat accumulation [9], but blocking Gas6 signaling using an Axl antagonist could reduce body-fat mass and body weight [13]. Interestingly, transgenic animals that ectopically express the Axl tyrosine kinase receptor also develop progressive obesity with elevated circulating proinflammatory cytokines and severe systemic insulin resistance [14]. This protein-array study also revealed higher levels of Axl mRNA in subcutaneous adipose tissue of obese humans than their lean control counterparts had. This indicates that the Axl receptor may be involved in the development of human obesity [15]. In addition, some studies in transgenic mice indicate that Gas6/Axl signaling might recruit macrophages and other immune cells into the adipose tissue resulting in the production and secretion of proinflammatory mediators. This suggests that the Gas6/Axl signaling might play a role in the pathogenesis of obesity-associated systemic inflammation [8, 16, 17]. Recent studies have indicated that systemic inflammation, a hallmark of childhood and adult obesity, is a pivotal mechanism linking obesity to insulin resistance and type 2 diabetes [18–21].

Although *GAS6 *gene polymorphisms are reported to be associated with stroke, acute coronary syndrome, and type 2 diabetes [22–24], to our knowledge, both *GAS6* and *AXL* gene polymorphisms associated with childhood obesity have not yet been identified. In order to address this issue, we conducted a community-based study to determine whether common variations in the *GAS6* and *AXL* genes correlate with adiposity, systemic inflammation, insulin resistance among adolescents.

## 2. Materials and Methods

### 2.1. Study Design and Sampling

The Taipei Children Heart Study-III was an epidemiological survey that investigated obesity and cardiovascular disease risk factors among adolescents in Taipei City during 2006. The sampling method and results have been previously described [25]. Briefly, the survey included junior high school students in Taipei City to collect a representative distribution of demographic, lifestyle, and biochemical characteristics to measure their risk for cardiovascular disease. After multistage sampling, researchers randomly selected 1283 Taipei adolescents. Those with autoimmune diseases, cancers, or active infection and those taking medications known to interfere with insulin or glucose metabolism were excluded. Excluding any missing data, 727 adolescents (358 boys and 369 girls) were included in the final analyses.

### 2.2. Data Collection

The institutional review board of the Tri-Service General Hospital approved these studies and obtained informed consent from both parents and adolescents. All the participants completed a structured questionnaire detailing their gender, age, puberty development, and lifestyle characteristics (including cigarette smoking, alcohol consumption, and physical activity). Based on their responses, the subjects were divided into young adolescents with history of smoking, those without, and those who currently smoke. The study divided alcohol consumption into 2 categories: present or no consumption. Physical activity was divided into 5 levels based on amount of exercise per week: less than 1 h, 1–3 h, 3–5 h, 5–7 h, and over 7 h. Survey questions concerning puberty onset included the development of the penis/testis and pubic hair for boys and development of breasts and pubic hair for girls. Pubertal status was evaluated according to the Tanner criteria [26].

### 2.3. Anthropometric Measurements

Body weight was measured of barefoot students wearing light indoor clothing and was rounded to the nearest 0.1 kg. Body height was recorded to the nearest 0.1 cm. Waist circumference (WC) was measured at the midway point between the inferior margin of the last rib and the crest of the ilium in a horizontal plane and was recorded to the nearest 0.1 cm. Hip circumference was measured at its widest point to the nearest 0.1 cm. Body mass index (BMI) was calculated as weight in kilograms divided by the square of height in meters.

### 2.4. Analytical Methods

To reduce extraneous variation between subjects, we collected blood samples from the students after 12 h fasting and who had consumed their usual diet for the previous 3 days. Children who had recently attended a holiday or family celebration were contacted for a blood sample several weeks after the event. Biochemical assays were performed within 2 weeks of blood collection and storage at −4°C. Plasma was stored at −70°C until used for biochemical analysis.

Plasma glucose concentrations were determined by the glucose oxidase method by using the Beckman Glucose Analyzer II (Beckman Instruments, Fullerton, CA). The intra- and interassay coefficients of variation (CVs) for glucose were 0.6% and 1.5%, respectively. Plasma insulin was measured using a commercial immunoradiometric kit (BioSource Europe, Nivelles, Belgium). The intra- and interassay CVs for insulin were 2.2% and 6.5%, respectively. Serum levels for high-sensitivity C-reactive protein (hsCRP) were measured using the Tina-Quant (Latex) high-sensitivity assay (Roche, Mannheim, Germany). The intra- and interassay CVs for hsCRP were 3.7% and 4.9%, respectively. Serum IL-6 concentrations were determined using a human high-sensitivity enzyme-linked immunosorbent assay (ELISA) (Innotest, Besancon, France). The intra- and interassay CVs for IL-6 were 1.5% and 5.3%, respectively. Serum TNF-*α* was measured with the Biotrak high-sensitivity human ELISA kit from Amersham Biosciences (Buckinghamshire, UK). The intra- and interassay CVs for TNF-*α* were 3.5% and 5.3%, respectively. All concentrations of the above biochemical variables are the means of 2 samples. Insulin resistance was assessed using the homeostasis model assessment (HOMA), in which the HOMA of insulin resistance (HOMA-IR) = fasting insulin (*μ*U/mL) × fasting glucose (mmol/L)/22.5 [27].

Gas6 protein concentration was measured using a sandwich ELISA and a polyclonal mouse anti-human Gas6 antibody (R&D Systems, Lille, France) as a catcher and a biotinylated goat antiserum as a detector (R&D Systems), using previously described methods [28]. The technique has been validated by Food and Drug Administration guidelines in a previous study (intra- and interassay CVs of 6.5% and 8.5%, resp.; mean recovery on 10 patients of 97%; lower limit of quantification 0.26 ng/mL) [29].

### 2.5. DNA Extraction and Genotype Analysis

DNA was isolated from buffy coats using the QIAamp DNA blood kit and following the manufacturer's instruction (Qiagen, Valencia, CA, USA). The qualities of isolated genomic DNAs were quantified using agarose gel electrophoresis, and the quantities were determined using a spectrophotometer. Genotyping was performed using quantitative real-time PCR. The SNP selection and primer design are described in a previous study [30]. SNPs rs8191973 and rs8191974 in *GAS6*, as well as rs4802113 and rs2304232 in *AXL*, were genotyped using a TaqMan assay with allele-specific probes on the ABI Prism 7900HT Sequence Detection System (Applied Biosystems, Foster City, CA, USA) according to the standardized laboratory protocols [24].

### 2.6. Statistical Methods

Descriptive results of continuous variables were expressed as means ± SD. Prior to statistical analysis, the normal distribution and homogeneity of the variables were evaluated using the Levene test for quality of variance, and the variables were then given a base logarithmic transformation if necessary. The parameters HOMA-IR, triglycerides, hsCRP, IL-6, and TNF-*α* were analyzed and tested for significance using a log scale. The studied adolescents were categorized into subgroups based on their *GAS6* rs8191973 genotype (CC, CG, and GG), *GAS6* rs8191974 genotype (GG, GA, and AA), *AXL* rs4802113 genotype (CC, CT, and TT), and *AXL* rs2304232 (AA, AG, and GG) with gender specification. The differences between anthropometric and biochemistry data across genotypes were analyzed using a general linear model after adjusting for age, Tanner stages, smoking status, drinking status, and physical activity. Chi-square tests were used to determine the genotype distributions for the Hardy-Weinberg equilibrium and to compare the proportions of abnormal anthropometric and biochemistry variables across genotypes. We tested different genetic inheritance models, and a recessive model was applied in the final analyses for *GAS6 *and *AXL*. To determine whether the *GAS6* and *AXL* SNPs are predictors of obesity and obesity-associated complications, logistic regression analysis was used to calculate the odds ratio (OR) and 95% confidence interval (CI) for each genotype and combined genotypes. A two-sided *P-*value of <0.05 was considered statistically significant. All statistical analyses were performed using PASW Statistics 18.0 software (SPSS Inc., Chicago, IL, USA).

## 3. Results

### 3.1. Subject Characteristics

In total, 727 adolescents (358 boys and 369 girls) were included in this study. The mean age of all adolescents in this study was 13.3 years (range, 12–15) and was similar between boys and girls. In general, boys had higher BMI (22.3 ± 4.0 versus 21.2 ± 3.3 kg/m^2^), WC (80.0 ± 10.1 versus 75.1 ± 8.1 cm), hsCRP (0.9 ± 1.3 versus 0.6 ± 0.9 mg/L), and glucose levels (93.8 ± 6.3 versus 91.5 ± 6.5 mg/dL) than the girls (all *P* < 0.001). However, girls had higher Tanner stages (3.2 ± 0.5 versus 3.0 ± 0.4) than the boys (*P* < 0.001). There was no statistically significant difference in the ages, TNF-*α*, IL-6, and insulin levels, and HOMA-IR between the boys and girls.

### 3.2. Genotype and Allele Frequencies

The genotype frequencies of the 4 polymorphisms are presented in Tables 1–4. All genotype frequencies were found to be within the Hardy-Weinberg equilibrium. The allele frequency for the least frequent allele in boys was 12.6, 22.1, 41.9, and 29.7%, and 13.4, 19.6, 32.7, and 33.5% in girls for the *GAS6* rs8191973, *GAS6* rs8191974, *AXL* rs4802113, and *AXL* rs2304232 polymorphisms, respectively. There was no significant difference in allele or genotype distribution between boys and girls at the 4 polymorphisms.

### 3.3. Association of *GAS6* Gene Polymorphisms with Adiposity, Inflammatory Markers, and HOMA-IR

No statistically significant association between anthropometric characteristics, biochemistry data, and the *GAS6 *rs8191973 genotypes was observed in the boys and girls (Table 1). However, there were significantly different hsCRP levels between GG, GA, and AA genotypes of *GAS6* rs8191974 in boys, regardless of their age, Tanner stages, smoking status, drinking status, or physical activity (Table 2). Moreover, boys with the GG genotype of *GAS6* rs8191974 had significantly higher BMI, WC, and hsCRP levels than those carrying the A allele. The *GAS6 *rs819174 genotypes were not significantly associated with any anthropometric characteristics and biochemistry in girls. The *P-*values of all comparisons between anthropometric and biochemistry data across *GAS6 *genotypes were presented in Supplemental Tables 1 and 2 available online at http://dx.doi.org/10.1155/2014/674069.

In addition, the association between circulating Gas6 protein levels and *GAS6* polymorphisms was investigated. We found that the *GAS6* rs8191973 or rs8191974 genotypes were not significantly associated with circulating Gas6 protein levels in both the sexes.

### 3.4. Association of *AXL* Gene Polymorphisms with Adiposity, Inflammatory Markers, and HOMA-IR

There were significantly different hsCRP levels between TT, TC, and CC genotypes of *AXL* rs4802113 in boys, independent of their age, Tanner stages, smoking status, drinking status, or physical activity (Table 3). In addition, boys with the GG genotype of *AXL *rs2304232 had significantly higher IL-6 and insulin levels and increased HOMA-IR than those carrying the A allele (Table 4). However, in girls, *AXL* rs4802113 or rs2304232 polymorphisms were not significantly associated with any anthropometric characteristics or biochemistry (Tables 3 and 4). The *P-*values of all comparisons between anthropometric and biochemistry data across *AXL *genotypes were presented in Supplemental Tables 3 and 4.

### 3.5. Association of *GAS6* and *AXL* Gene Polymorphisms with Elevated Adiposity, Inflammatory Markers Levels, and HOMA-IR

Boys with the GG genotype of *GAS6* rs8191973 were 1.87-fold more likely to have higher hsCRP levels than the C allele carriers. Even after adjusting for age, Tanner stage, smoking status, drinking status, and physical activity, a significant relationship between the GG genotype of *GAS6* rs8191973 and higher hsCRP levels was still observed in boys (Table 5). Moreover, boys with the GG genotype of *GAS6* rs8191974 exhibited a 1.40-fold greater risk for developing high BMI, a 1.58-fold greater risk for developing high WC, and a 2.68-fold greater risk to have higher IL-6 levels than the A allele carriers. Even after adjusting for all possible confounding factors including age, Tanner stage, smoking/drinking status, and physical activity, the relationship between the GG genotype of *GAS6* rs8191974, higher BMI/WC, and higher IL-6 levels still remained significant in boys. However, the *AXL *rs4802113 or rs2304232 polymorphisms showed no significant association with abnormal adiposity, inflammatory markers, and HOMA-IR in boys or girls (see Supplemental Table 5).

### 3.6. Combined Effect of the *GAS6* and *AXL* Polymorphisms on High Adiposity, Inflammatory Marker Levels, and HOMA-IR

**Figure 1:**
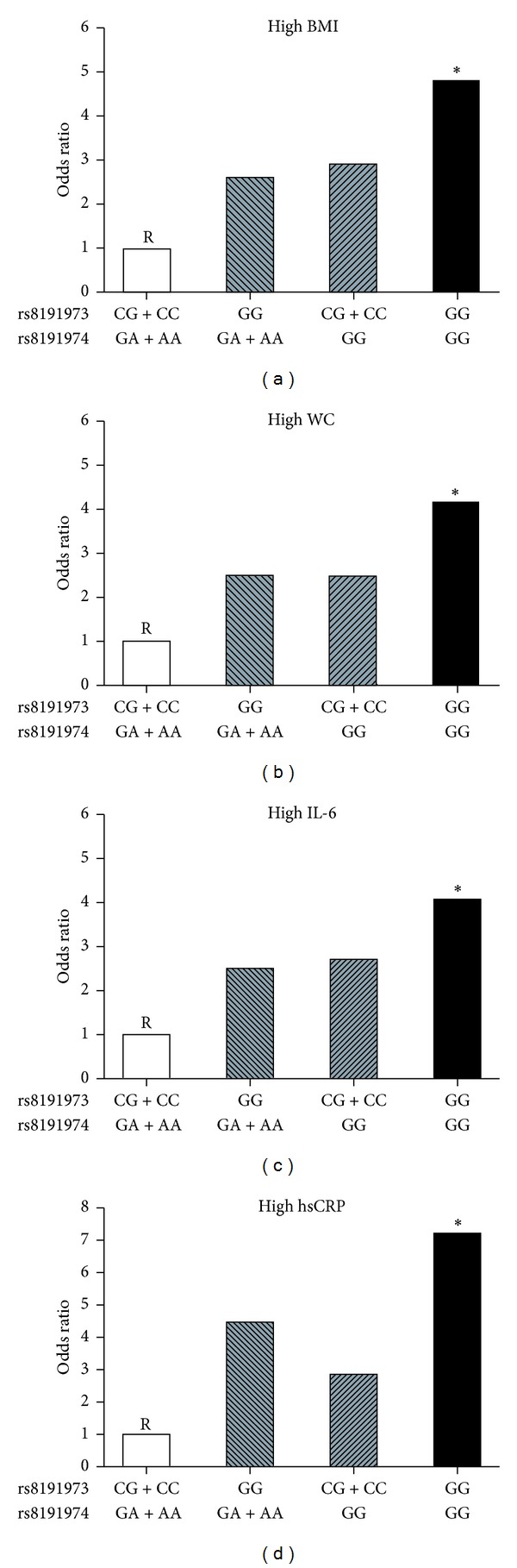
Combined effect of *GAS6* rs8191973 and rs8191974 polymorphisms on the risk of abnormal variables in boys. *P < 0.05; R = reference group.

Logistic regression analyses were applied to evaluate whether the combination of the *GAS6* rs8191974 and rs8191973 polymorphisms is a stronger risk factor for high adiposity, inflammatory markers levels, and HOMA-IR than when alone. The combined effects of the 2 *GAS6* gene polymorphisms in the risk of high BMI, WC, IL-6, and hsCRP levels are shown in Figure 1. After adjusting for the relevant confounding factors, we still observed that boys with the GG genotype of *GAS6* rs8191973 and the GG genotype of *GAS6* rs8191974 exhibited a 4–7-fold higher risk of high BMI, WC, IL-6, and hsCRP levels than the individuals with both the C allele of the *GAS6* rs8191973 and the A allele of the *GAS6* rs8191974 did (OR = 4.92, 95% CI: 1.08–23.6, *P* = 0.018; OR = 4.18, 95% CI: 1.05–22.5, *P* = 0.016; OR = 4.08, 95% CI: 1.06–28.56, *P* = 0.015; OR = 7.22, 95% CI: 1.46–35.72, *P* = 0.010, resp.). However, for girls, there was no statistically significant association between the combination of the *GAS6* rs8191974 and rs8191973 polymorphisms and abnormal variables.

In addition, we evaluated the combined effect of the *GAS6* rs8191973 or rs8191974 marker with *AXL* gene polymorphisms and its association with risk of high adiposity, inflammatory marker, and HOMA-IR. However, combinations of* GAS6 *markers with *AXL* gene polymorphisms were not found to be significantly associated with any abnormal variables in both boys and girls (data not shown).

## 4. Discussion

In this study, a strong association between *GAS6* and *AXL* polymorphisms with body adiposity, systemic inflammation, and insulin resistance was identified among boys. The risk of possessing high adiposity and inflammatory markers levels was higher in boys carrying the GG genotype with *GAS6* rs8191973 or rs8191974 than the noncarriers. Moreover, the combination of both *GAS6* polymorphisms had an additive effect on the development of obesity and obesity-associated inflammation in boys. These data strongly suggest that *GAS6* and *AXL* genes play a role in the pathogenesis of childhood obesity and its associated complications.

*GAS6* was originally identified as a gene that is expressed in fibroblasts and increases with serum starvation and contact inhibition [6]; Gas6 is also a potential growth factor for fibroblasts [11]. Maquoi and colleagues demonstrated that when fed with a high-fat diet, *GAS6*-deficient mice had significantly less fat than their wild-type counterparts [9]. The authors also reported the expression of Gas6 and its 3 receptors (Tyro-3, Axl, Mer) in murine adipose tissues, thus suggesting that Gas6 may act in an autocrine and/or paracrine manner to promote murine adipose tissue development [9]. Previous experiments in transgenic mice demonstrate that Gas6 might also induce obesity-associated inflammation via recruiting immune cells into the adipose tissue to producing and secreting proinflammatory cytokines [8, 16, 17]. Our recent clinical study found that circulating Gas6 protein levels are associated with adiposity and inflammatory markers in overweight/obese adolescents [5]. In this study, *GAS6* is further implicated as a candidate susceptibility gene for obesity and systemic inflammation. However, the association between *GAS6* genotypes and circulating Gas6 protein levels was not observed among adolescents. We hypothesize that *GAS6* polymorphisms could affect the biology of the Gas6 protein itself rather than its transcription or process rate, thus influencing adiposity regulation and systemic inflammation. To validate this, further studies regarding the association between Gas6 protein biology and *GAS6 *polymorphisms are required.

Recent studies demonstrated that Gas6/TAM signaling is involved in releasing inflammatory cytokines (such as IL-6 and hsCRP) in diverse human diseases [23, 31, 32]. In addition, the Gas6/TAM signaling is also known to be involved in several inflammation-related systems, including maturation of immune cells [33], endothelial activation [7], and immunoregulation [34]. Our present study found that the GG genotype of *GAS6* rs8191973 and the GG genotype of *GAS6* rs8191974 are strongly associated with higher circulating IL-6 and hsCRP levels in boys. Therefore, the *GAS6* polymorphisms presumably influence Gas6/TAM signaling and could further activate inflammatory reactions and result in releasing circulating IL-6 and hsCRP. However, the comprehensive effects of the *GAS6* polymorphisms in regulation of inflammatory cytokines still remain to be determined by more researches.

Interestingly, a previous study found that the A allele or the AA genotype of *GAS6* rs8191974 is associated with decreased risk of stroke [22]. Moreover, the A allele and the AA genotype are also thought to be related to a lower risk of developing acute coronary syndrome or type 2 diabetes, suggesting that this genotype exhibits protective activities against developing acute coronary syndrome and type 2 diabetes [23, 24]. We also observed similar results in those with the A allele or AA genotype of* GAS6 *rs8191974. These subjects exhibited a lower risk for developing obesity and systemic inflammation than those with the GG genotypes. Together, these findings suggest that the *GAS6* rs8191974 polymorphisms play an important role in the development of obesity and obesity-associated complications (e.g., type 2 diabetes, cerebrovascular, and cardiovascular diseases). The protective role of the AA genotype of *GAS6* rs8191974 against the developing childhood obesity and obesity-associated complications requires further study.

The Axl protein is a membrane-bound receptor that belongs to the TAM family of receptor tyrosine kinases. Gas6 and protein S are the known ligands of the TAM receptor family [35]. Axl exhibits the highest affinity for Gas6 as compared to the other members of the TAM family, whereas protein S predominantly binds Mer and Tyro-3 [36]. Gas6/Axl signaling has been shown to be involved in the pathogenesis of obesity and systemic inflammation [13–15]. However, our study demonstrates that *AXL* polymorphisms are associated with systemic inflammation rather than childhood obesity. Moreover, the combination of* GAS6* and *AXL* gene polymorphisms is not significantly associated with any variables in adiposity among adolescents. Our findings indicated that *AXL* gene polymorphisms might not play a significant role in childhood obesity. Recently, Scroyen and colleagues [37] have published similar findings indicating that deficiency in a single Axl receptor did not significantly affect adipogenesis or adipose tissue development in mice. This is because an Axl deficiency can be partially compensated by other TAM family members (Tyro-3 and Mer) via Gas6 interaction. Axl may not be the only TAM receptor through which Gas6 could modulate adipogenesis. Further studies are needed to investigate the effect of Tyro-3 and Mer receptors on the development of childhood obesity.

In addition, our present study also indicates that gender disparity exists regarding the effects of the *GAS6 *polymorphisms on anthropometric characteristics and inflammatory markers. We found no significant difference in genotype frequencies between boys and girls; however, the effects of the *GAS6* polymorphisms, individually or combined, only manifest in boys. The *GAS6* gene contains an estrogen response element in its promoter and is upregulated by estrogen via an activated estrogen receptor in mammary epithelial cells [38]. Moreover, androgen was reported to directly regulate *GAS6* transcription via the androgen receptor [39]. Therefore, we hypothesized that the gender-specific effect of the *GAS6* polymorphisms on childhood obesity might be due to a disparity in sex hormone distributions. This has been previously reported to be associated with *GAS6* expression and body composition [40, 41].

Despite these results, our study does have certain limitations. First, this was a cross-sectional study, as such we might not be able to assess *GAS6* polymorphisms on weight dynamics and the development of obesity-associated complications throughout life. Furthermore, longitudinal studies are required to confirm our results. Second, because of the limitations of our questionnaire, we were not able to comprehensively estimate every adolescent's daily intake. The impact of dietary energy intake on genetic susceptibility also requires further investigation to better understand any confounding effect.

In conclusion, we indicate an association between the *GAS6* and *AXL* polymorphisms with adiposity, circulating inflammatory markers, and insulin resistance of adolescents, especially in boys. Moreover, the GG genotype of *GAS6* rs8191973 or rs8191974 strongly correlates with susceptibility to develop obesity and systemic inflammation in boys. Nonetheless, these results together with those from studies in cellular and animal models encourage the study of the Gas6/TAM system in childhood obesity and its potential complications and further support the hypothesis that modulation of Gas6 activity may indeed provide an important intervention point for future therapies.

## Supplementary Material

In order to explore the effects of *GAS6/ AXL* gene polymorphisms on adiposity, systemic inflammation, and insulin resistance, we genotyped 727 adolescents' *GAS6* rs8191973, *GAS6* rs8191974, *AXL* rs4802113, and *AXL* rs2304232 polymorphisms from the Taipei Children Heart Study-III. The studied adolescents were categorized into subgroups based on their *GAS6* rs8191973 genotype (CC, CG, and GG), *GAS6* rs8191974 genotype (GG, GA, and AA), *AXL* rs4802113 genotype (CC, CT, and TT), and *AXL* rs2304232 (AA, AG, and GG) with gender specification. All comparisons between anthropometric and biochemistry data across *GAS6* and *AXL* polymorphisms were presented in Supplemental Tables 1-4. To determine whether the *AXL* SNPs are predictors of obesity and obesity-associated complications, logistic regression analysis was used to calculate the odds ratio and 95% confidence interval for each genotype. The results were present in Supplemental Table 5.

## Figures and Tables

**Table 1:** Anthropometric and biochemical data with different *GAS6 *rs8191973 genotypes among boys and girls.

	Boys			Girls		
	GG	GC	CC	GG	GC	CC
	(*n* = 277)	(*n* = 72)	(*n* = 9)	(*n* = 277)	(*n* = 85)	(*n* = 7)
BMI (kg/m^2^)	22.4 ± 4.0	21.7 ± 4.0	24.2 ± 2.8	21.1 ± 3.2	21.6 ± 3.7	21.1 ± 2.0
WC (cm)	80.1 ± 10.5	79.2 ± 10.4	84.7 ± 8.6	75.2 ± 7.8	76.5 ± 9.2	78.6 ± 6.8
hsCRP (mg/L)	0.8 ± 1.3	1.0 ± 1.3	1.0 ± 1.7	0.6 ± 0.9	0.6 ± 0.7	0.3 ± 0.2
TNF-*α* (pg/mL)	26.8 ± 2.9	23.5 ± 4.1	25.3 ± 6.2	22.7 ± 4.7	27.3 ± 5.4	25.1 ± 7.5
IL-6 (pg/mL)	3.2 ± 2.1	3.5 ± 3.1	3.9 ± 3.0	3.3 ± 3.1	3.0 ± 1.4	2.8 ± 1.1
Glucose (mg/dL)	94.1 ± 6.4	92.7 ± 5.8	93.7 ± 4.1	91.7 ± 6.4	91.1 ± 7.3	88.3 ± 3.5
Insulin (*μ*U/mL)	15.4 ± 8.8	13.8 ± 8.3	17.1 ± 6.3	14.1 ± 7.5	15.7 ± 8.0	15.9 ± 5.1
HOMA-IR	3.6 ± 2.2	3.2 ± 2.1	4.0 ± 1.6	3.2 ± 1.8	3.6 ± 1.9	3.4 ± 1.0
Gas6 (ng/mL)	12.1 ± 3.3	14.1 ± 3.7	12.9 ± 3.7	12.6 ± 3.9	12.2 ± 3.1	11.8 ± 3.1

Data are expressed as mean ± SD.

BMI: body mass index; WC: waist circumference; HOMA-IR: homeostasis model assessment of insulin resistance; hsCRP: high-sensitivity C-reactive protein; TNF-*α*: tumor necrosis factor-*α*; IL-6: interleukin-6.

**Table 2:** Anthropometric and biochemical data with different *GAS6 *rs8191974 genotypes among boys and girls.

	Boys			Girls		
	GG	GA	AA	GG	GA	AA
	(*n* = 213)	(*n* = 132)	(*n* = 13)	(*n* = 242)	(*n* = 109)	(*n* = 18)
BMI (kg/m^2^)	22.5 ± 4.2^b^	22.0 ± 3.6^b^	20.7 ± 3.3^b^	21.2 ± 3.3	21.3 ± 3.4	20.3 ± 2.3
WC (cm)	80.6 ± 11.0^b^	79.3 ± 9.5^b^	76.7 ± 9.4^b^	75.8 ± 8.1	75.2 ± 8.2	74.6 ± 8.3
hsCRP (mg/L)	0.8 ± 1.2^a,b^	1.1 ± 1.6^a,b^	0.4 ± 0.2^a,b^	0.6 ± 1.0	0.5 ± 0.5	0.9 ± 1.3
TNF-*α* (pg/mL)	26.7 ± 8.2	25.5 ± 10.1	23.8 ± 4.6	24.5 ± 9.3	22.9 ± 6.5	27.9 ± 4.3
IL-6 (pg/mL)	3.4 ± 2.8	3.3 ± 3.2	2.5 ± 1.3	3.2 ± 2.8	3.1 ± 3.0	2.9 ± 0.7
Glucose (mg/dL)	94.0 ± 6.3	93.4 ± 6.4	95.3 ± 3.6	91.8 ± 6.7	90.6 ± 6.3	92.1 ± 5.7
Insulin (*μ*U/mL)	14.9 ± 8.7	15.3 ± 9.0	15.0 ± 8.2	15.0 ± 8.1	13.9 ± 6.6	11.7 ± 4.2
HOMA-IR	3.5 ± 2.2	3.6 ± 2.2	3.5 ± 2.0	3.4 ± 2.0	3.1 ± 1.5	2.7 ± 1.1
Gas6 (ng/mL)	13.1 ± 3.7	13.2 ± 3.7	13.0 ± 3.1	11.8 ± 3.1	12.1 ± 3.0	1.7 ± 2.2

Data are expressed as mean ± SD.

BMI: body mass index; WC: waist circumference; HOMA-IR: homeostasis model assessment of insulin resistance; hsCRP: high-sensitivity C-reactive protein; TNF-*α*: tumor necrosis factor-*α*; IL-6: interleukin-6.

^a^GG versus GA versus AA, *P* < 0.05; ^b^GG versus (GA + AA), *P *< 0.05. All comparisons were analyzed using a general linear model after adjusting for age, Tanner stages, smoking status, drinking status, and physical activity.

**Table 3:** Anthropometric and biochemical data with different *AXL *rs4802113 genotypes among boys and girls.

	Boys			Girls		
	TT	TC	CC	TT	TC	CC
	(*n* = 123)	(*n* = 170)	(*n* = 65)	(*n* = 108)	(*n* = 181)	(*n* = 80)
BMI (kg/m^2^)	22.0 ± 3.7	22.6 ± 3.8	21.9 ± 4.7	21.4 ± 3.6	21.1 ± 3.2	21.2 ± 3.0
WC (cm)	79.4 ± 10.3	80.8 ± 10.0	79.0 ± 11.9	75.8 ± 9.0	75.2 ± 7.7	76.1 ± 7.9
hsCRP (mg/L)	0.8 ± 1.2^a^	1.0 ± 1.4^a^	0.8 ± 1.3^a^	0.6 ± 0.8	0.6 ± 0.9	0.6 ± 0.9
TNF-*α* (pg/mL)	24.8 ± 5.5	26.2 ± 6.5	28.2 ± 10.1	24.0 ± 7.3	25.7 ± 8.3	26.6 ± 8.3
IL-6 (pg/mL)	3.0 ± 2.8	3.4 ± 2.7	3.7 ± 3.5	3.5 ± 3.0	2.9 ± 2.0	3.3 ± 2.9
Glucose (mg/dL)	93.4 ± 6.1	94.3 ± 6.0	93.4 ± 7.1	92.0 ± 6.9	91.0 ± 6.5	91.8 ± 6.2
Insulin (*μ*U/mL)	14.8 ± 8.1	15.6 ± 9.7	14.5 ± 7.9	15.2 ± 7.3	14.1 ± 7.8	14.7 ± 7.4
HOMA-IR	3.4 ± 2.0	3.7 ± 2.3	3.4 ± 2.0	3.5 ± 1.7	3.2 ± 1.9	3.3 ± 1.7

Data are expressed as mean ± SD.

BMI: body mass index; WC: waist circumference; HOMA-IR: homeostasis model assessment of insulin resistance; hsCRP: high-sensitivity C-reactive protein; TNF-*α*: tumor necrosis factor-*α*; IL-6: interleukin-6.

^a^TT versus TC versus CC, *P* < 0.05. All comparisons were analyzed using a general linear model after adjusting for age, Tanner stages, smoking status, drinking status, and physical activity.

**Table 4:** Anthropometric and biochemical data with different *AXL *rs2304232 genotypes among boys and girls.

	Boys			Girls		
	AA	AG	GG	AA	AG	GG
	(*n* = 180)	(*n* = 143)	(*n* = 35)	(*n* = 165)	(*n* = 161)	(*n* = 43)
BMI (kg/m^2^)	22.3 ± 3.7	22.2 ± 3.9	22.4 ± 5.4	21.4 ± 3.5	21.0 ± 3.3	21.2 ± 2.5
WC (cm)	79.8 ± 10.1	80.3 ± 10.0	79.5 ± 13.8	75.7 ± 8.6	75.5 ± 7.9	75.0 ± 7.3
hsCRP (mg/L)	0.9 ± 1.5	0.8 ± 1.0	0.8 ± 1.5	0.5 ± 0.8	0.5 ± 0.8	0.8 ± 1.4
TNF-*α* (pg/mL)	26.9 ± 6.0	25.2 ± 7.0	24.7 ± 7.7	24.7 ± 7.5	25.4 ± 8.4	28.1± 9.5
IL-6 (pg/mL)	3.1 ± 3.0^a^	3.2 ± 3.1^a^	4.8 ± 4.0^a^	3.2 ± 3.1	3.0 ± 1.8	3.7 ± 3.5
Glucose (mg/dL)	93.7 ± 5.8	94.0 ± 6.2	93.4 ± 8.4	91.3 ± 6.5	91.5 ± 6.7	92.0 ± 6.0
Insulin (*μ*U/mL)	15.4 ± 9.3^a^	14.5 ± 8.2^a^	15.9 ± 8.6^a^	15.1 ± 7.5	14.0 ± 7.9	14.1 ± 6.1
HOMA-IR	3.6 ± 2.3^a^	3.4 ± 2.0^a^	3.7 ± 2.3^a^	3.4 ± 1.8	3.2 ± 1.9	3.2 ± 1.4

Data are expressed as mean ± SD.

BMI: body mass index; WC: waist circumference; HOMA-IR: homeostasis model assessment of insulin resistance; hsCRP: high-sensitivity C-reactive protein; TNF-*α*: tumor necrosis factor-*α*; IL-6: interleukin-6.

^a^(AA + AG) versus GG, *P* < 0.05. All comparisons were analyzed using a general linear model after adjusting for age, Tanner stages, smoking status, drinking status, and physical activity.

**Table 5:** Logistic regression analyses of different *GAS6* SNP on abnormal variables among adolescents.

Variables^b^	*GAS6* rs8191973		*GAS6* rs8191974	
	GG OR (95% CI)^a^		GG OR (95% CI)^a^	
	Unadjusted	Adjusted^c^	Unadjusted	Adjusted^c^
Boys				
High BMI	1.12 (0.47–2.68)	1.26 (0.34–4.76)	1.40 (1.06–2.99)^d^	1.85 (1.04–3.23)^d^
High WC	0.82 (0.38–1.76)	1.11 (0.39–5.26)	1.58 (1.18–2.01)^d^	1.68 (1.08–3.25)^d^
High hsCRP	1.87 (1.25–2.87)^d^	2.53 (1.03–6.24)^d^	1.88 (0.68–3.25)	1.92 (0.93–3.96)
High TNF-*α*	4.26 (0.99–18.37)	3.34 (0.42–24.98)	0.93 (0.43–1.98)	0.99 (0.33–2.94)
High IL-6	2.09 (0.92–4.82)	2.27 (0.98–5.26)	2.68 (1.32–5.20)^d^	2.56 (1.33–5.00)^d^
High HOMA-IR	1.41 (0.56–3.52)	1.14 (0.31–4.17)	1.28 (0.61–2.67)	2.35 (0.73–7.69)
Girls				
High BMI	0.76 (0.36–1.61)	0.57 (0.22–1.45)	1.09 (0.53–2.26)	1.10 (0.43–2.86)
High WC	0.60 (0.30–1.23)	0.44 (0.17–1.11)	1.26 (0.60–2.64)	1.57 (0.55–4.55)
High hsCRP	0.90 (0.41–1.96)	1.04 (0.39–2.78)	1.19 (0.58–2.44)	1.05 (0.44–2.50)
High TNF-*α*	0.91 (0.41–2.04)	0.69 (0.24–1.96)	1.00 (0.47–2.13)	0.85 (0.31–2.33)
High IL-6	1.37 (0.61–3.09)	0.90 (0.34–2.50)	1.42 (0.69–2.95)	1.68 (0.06–4.55)
High HOMA-IR	0.76 (0.33–1.72)	0.69 (0.25–1.92)	1.48 (0.64–3.41)	2.20 (0.70–7.14)

^a^Under a recessive model (using heterozygotes and minor homozygotes as the reference for each SNP). ^b^Abnormal variables were determined using an age- and gender-specific 90th percentile cut-off point.

^c^Adjusting for age, Tanner stage, cigarette smoking, alcohol drinking, and physical activity.

^d^ *P* < 0.05.

OR: odds ratio; CI: confidence index; BMI: body mass index; WC: waist circumference; HOMA-IR: homeostasis model assessment of insulin resistance; hsCRP: high-sensitivity C-reactive protein; TNF-*α*: tumor necrosis factor-*α*; IL-6: interleukin-6.
