# Supplementary material for: Effect of GAS6 and AXL Gene Polymorphisms on Adiposity, Systemic Inflammation, and Insulin Resistance in Adolescents
Source: Int J Endocrinol. 2014 Feb 18;2014:674069. doi: 10.1155/2014/674069 (PMC3948192; doi:10.1155/2014/674069)
Supplement: Supplementary file 1 — In order to explore the effects of GAS6/ AXL gene polymorphisms on adiposity, systemic inflammation, and insulin resistance, we genotyped 727 adolescents' GAS6 rs8191973, GAS6 rs8191974, AXL rs4802113, and AXL rs2304232 polymorphisms from the Taipei Children Heart Study-III. The studied adolescents were categorized into subgroups based on their GAS6 rs8191973 genotype (CC, CG, and GG), GAS6 rs8191974 genotype (GG, GA, and AA), AXL rs4802113 genotype (CC, CT, and TT), and AXL rs2304232 (AA, AG, and GG) with gender specification. All comparisons between anthropometric and biochemistry data across GAS6 and AXL polymorphisms were presented in Supplemental Tables 1-4. To determine whether the AXL SNPs are predictors of obesity and obesity-associated complications, logistic regression analysis was used to calculate the odds ratio and 95% confidence interval for each genotype. The results were present in Supplemental Table 5. [file 674069.f1.pdf]

Supplemental Data

Supplemental Table 1. Anthropometric and biochemical data among all adolescents with different *GAS6* rs8191973 genotypes

	Genotypes			GLM test[a]		
	GG	GC	CC	p1	p2	p3
Boys						
Numbers (%)	277 (77.4)	72 (20.1)	9 (2.5)			
BMI (kg/m^2)	22.4 ± 4.0	21.7 ± 4.0	24.2 ± 2.8	0.165	0.483	0.318
WC (cm)	80.1 ± 10.5	79.2 ± 10.4	84.7 ± 8.6	0.325	0.858	0.221
hsCRP (mg/L)	0.8 ± 1.3	1.0 ± 1.3	1.0 ± 1.7	0.468	0.218	0.495
TNF-α (pg/mL)	26.8 ± 2.9	23.5 ± 4.1	25.3 ± 6.2	0.580	0.317	0.448
IL-6 (pg/mL)	3.2 ± 2.1	3.5 ± 3.1	3.9 ± 3.0	0.863	0.648	0.894
Glucose (mg/dL)	94.1 ± 6.4	92.7 ± 5.8	93.7 ± 4.1	0.218	0.091	0.395
Insulin (μU/mL)	15.4 ± 8.8	13.8 ± 8.3	17.1 ± 6.3	0.298	0.250	0.511
HOMA-IR	3.6 ± 2.2	3.2 ± 2.1	4.0 ± 1.6	0.253	0.197	0.438
Gas6 (ng/mL)	12.1 ± 3.3	14.1 ± 3.7	12.9 ± 3.7	0.524	0.310	0.785
Girls						
Numbers (%)	277 (75.1)	85 (23.0)	7 (1.9)			
BMI (kg/m^2)	21.1 ± 3.2	21.6 ± 3.7	21.1 ± 2.0	0.208	0.098	0.843
WC (cm)	75.2 ± 7.8	76.5 ± 9.2	78.6 ± 6.8	0.262	0.122	0.899
hsCRP (mg/L)	0.6 ± 0.9	0.6 ± 0.7	0.3 ± 0.2	0.817	0.706	0.689
TNF-α (pg/mL)	22.7 ± 4.7	27.3 ± 5.4	25.1 ± 7.5	0.401	0.183	0.917
IL-6 (pg/mL)	3.3 ± 3.1	3.0 ± 1.4	2.8 ± 1.1	0.964	0.790	0.913
Glucose (mg/dL)	91.7 ± 6.4	91.1 ± 7.3	88.3 ± 3.5	0.401	0.328	0.250
Insulin (μU/mL)	14.1 ± 7.5	15.7 ± 8.0	15.9 ± 5.1	0.317	0.129	0.671
HOMA-IR	3.2 ± 1.8	3.6 ± 1.9	3.4 ± 1.0	0.466	0.217	0.828

Gas6 (ng/mL)	12.6 ± 3.9	12.2 ± 3.1	11.8 ± 3.1	0.446	0.289	0.684

Abbreviations: GLM, general linear model; BMI, body mass index; WC, waist circumference; HOMA-IR, homeostasis model assessment of insulin resistance; hsCRP, high-sensitivity C-reactive protein; TNF-α, tumor necrosis factor-α; IL-6, interleukin-6.

[a]p1: GG vs. GC vs. CC; p2: GG vs. (GC+CC), p3: (GG+GC) vs. CC, after adjusting for age, Tanner stage, cigarette smoking, alcohol drinking, and physical activity.

Supplemental Table 2. Anthropometric and biochemical data among all adolescents with different *GAS6* rs8191974 genotypes

	Genotype			GLM test[a]		
	GG	GA	AA	p1	p2	p3
Boys						
Numbers (%)	213 (59.5)	132 (36.9)	13 (3.6)			
BMI (kg/m^2)	22.5 ± 4.2	22.0 ± 3.6	20.7 ± 3.3	0.131	**0.035**	0.776
WC (cm)	80.6 ± 11.0	79.3 ± 9.5	76.7 ± 9.4	0.150	**0.037**	0.950
hsCRP (mg/L)	0.8 ± 1.2	1.1 ± 1.6	0.4 ± 0.2	**0.033**	**0.042**	0.473
TNF-α (pg/mL)	26.7 ± 8.2	25.5 ± 10.1	23.8 ± 4.6	0.951	0.919	0.789
IL-6 (pg/mL)	3.4 ± 2.8	3.3 ± 3.2	2.5 ± 1.3	0.635	0.352	0.663
Glucose (mg/dL)	94.0 ± 6.3	93.4 ± 6.4	95.3 ± 3.6	0.625	0.928	0.361
Insulin (μU/mL)	14.9 ± 8.7	15.3 ± 9.0	15.0 ± 8.2	0.187	0.868	0.070
HOMA-IR	3.5 ± 2.2	3.6 ± 2.2	3.5 ± 2.0	0.169	0.915	0.064
Gas6 (ng/mL)	13.1 ± 3.7	13.2 ± 3.7	13.0 ± 3.1	0.183	0.361	0.075

Girls						
Numbers (%)	242 (65.6)	109 (29.5)	18 (4.9)			
BMI (kg/m^2)	21.2 ± 3.3	21.3 ± 3.4	20.3 ± 2.3	0.759	0.585	0.520
WC (cm)	75.8 ± 8.1	75.2 ± 8.2	74.6 ± 8.3	0.590	0.335	0.954
hsCRP (mg/L)	0.6 ± 1.0	0.5 ± 0.5	0.9 ± 1.3	0.241	0.631	0.161
TNF-α (pg/mL)	24.5 ± 9.3	22.9 ± 6.5	27.9 ± 4.3	0.857	0.590	0.769
IL-6 (pg/mL)	3.2 ± 2.8	3.1 ± 3.0	2.9 ± 0.7	0.364	0.156	0.606
Glucose (mg/dL)	91.8 ± 6.7	90.6 ± 6.3	92.1 ± 5.7	0.820	0.643	0.786
Insulin (μU/mL)	15.0 ± 8.1	13.9 ± 6.6	11.7 ± 4.2	0.088	0.078	0.137
HOMA-IR	3.4 ± 2.0	3.1 ± 1.5	2.7 ± 1.1	0.098	0.081	0.157
Gas6 (ng/mL)	11.8 ± 3.1	12.1 ± 3.0	1.7 ± 2.2	0.333	0.355	0.406

Abbreviations: GLM, general linear model; BMI, body mass index; WC, waist circumference; HOMA-IR, homeostasis model assessment of insulin resistance; hsCRP, high-sensitivity C-reactive protein; TNF-α, tumor necrosis factor-α; IL-6, interleukin-6.

Bold indicates significant comparisons (*P* < 0.05).

[a]p1: GG vs. GA vs. AA; p2: GG vs. (GA+AA), p3: (GG+GA) vs. AA, after adjusting for age, Tanner stage, cigarette smoking, alcohol drinking, and physical activity.

Supplemental Table 3. Anthropometric and biochemical data among all adolescents with different *AXL* rs4802113 genotypes

	Genotype			GLM test[a]		
	TT	TC	CC	p1	p2	p3
Boys						
Numbers (%)	123 (34.4)	170 (47.5)	65 (18.1)			
BMI (kg/m^2)	22.0 ± 3.7	22.6 ± 3.8	21.9 ± 4.7	0.467	0.409	0.255
WC (cm)	79.4 ± 10.3	80.8 ± 10.0	79.0 ± 11.9	0.496	0.473	0.260
hsCRP (mg/L)	0.8 ± 1.2	1.0 ± 1.4	0.8 ± 1.3	**0.036**	0.077	0.242
TNF-α (pg/mL)	24.8 ± 5.5	26.2 ± 6.5	28.2 ± 10.1	0.587	0.474	0.348
IL-6 (pg/mL)	3.0 ± 2.8	3.4 ± 2.7	3.7 ± 3.5	0.679	0.847	0.381
Glucose (mg/dL)	93.4 ± 6.1	94.3 ± 6.0	93.4 ± 7.1	0.616	0.546	0.591
Insulin (μU/mL)	14.8 ± 8.1	15.6 ± 9.7	14.5 ± 7.9	0.393	0.234	0.304
HOMA-IR	3.4 ± 2.0	3.7 ± 2.3	3.4 ± 2.0	0.388	0.221	0.317
Girls						
Numbers (%)	108 (29.3)	181 (49.1)	80 (21.6)			
BMI (kg/m^2)	21.4 ± 3.6	21.1 ± 3.2	21.2 ± 3.0	0.621	0.815	0.330
WC (cm)	75.8 ± 9.0	75.2 ± 7.7	76.1 ± 7.9	0.745	0.550	0.788
hsCRP (mg/L)	0.6 ± 0.8	0.6 ± 0.9	0.6 ± 0.9	0.933	0.877	0.786
TNF-α (pg/mL)	24.0 ± 7.3	25.7 ± 8.3	26.6 ± 8.3	0.859	0.908	0.633
IL-6 (pg/mL)	3.5 ± 3.0	2.9 ± 2.0	3.3 ± 2.9	0.152	0.105	0.614
Glucose (mg/dL)	92.0 ± 6.9	91.0 ± 6.5	91.8 ± 6.2	0.323	0.919	0.163
Insulin (μU/mL)	15.2 ± 7.3	14.1 ± 7.8	14.7 ± 7.4	0.693	0.995	0.415
HOMA-IR	3.5 ± 1.7	3.2 ± 1.9	3.3 ± 1.7	0.620	0.973	0.359

Abbreviations: GLM, general linear model; BMI, body mass index; WC, waist circumference; HOMA-IR, homeostasis model assessment of insulin resistance; hsCRP, high-sensitivity C-reactive protein; TNF-α, tumor necrosis factor-α; IL-6, interleukin-6.

Bold indicates significant comparisons (*P* < 0.05).

[a]p1: TT vs. TC vs. CC; p2: TT vs. (TC+CC); p3: (TT+TC) vs. CC, after adjusting for age, Tanner stage, cigarette smoking, alcohol drinking, and physical activity.

Supplemental Table 4. Anthropometric and biochemical data among all adolescents with different *AXL* rs2304232 genotypes

	Genotype			GLM test[a]		
	AA	AG	GG	p1	p2	p3
Boys						
Numbers (%)	180 (50.2)	143 (40.0)	35 (9.8)			
BMI (kg/m^2)	22.3 ± 3.7	22.2 ± 3.9	22.4 ± 5.4	0.176	0.183	0.101
WC (cm)	79.8 ± 10.1	80.3 ± 10.0	79.5 ± 13.8	0.267	0.150	0.252
hsCRP (mg/L)	0.9 ± 1.5	0.8 ± 1.0	0.8 ± 1.5	0.558	0.628	0.286
TNF-α (pg/mL)	26.9 ± 6.0	25.2 ± 7.0	24.7 ± 7.7	0.739	0.440	0.891
IL-6 (pg/mL)	3.1 ± 3.0	3.2 ± 3.1	4.8 ± 4.0	0.078	0.938	**0.033**
Glucose (mg/dL)	93.7 ± 5.8	94.0 ± 6.2	93.4 ± 8.4	0.642	0.870	0.350
Insulin (μU/mL)	15.4 ± 9.3	14.5 ± 8.2	15.9 ± 8.6	0.103	0.425	**0.033**
HOMA-IR	3.6 ± 2.3	3.4 ± 2.0	3.7 ± 2.3	0.094	0.445	**0.030**
Girls						
Numbers (%)	165 (44.7)	161 (43.6)	43 (11.7)			

BMI (kg/m^2)	21.4 ± 3.5	21.0 ± 3.3	21.2 ± 2.5	0.893	0.649	0.791
WC (cm)	75.7 ± 8.6	75.5 ± 7.9	75.0 ± 7.3	0.640	0.626	0.525
hsCRP (mg/L)	0.5 ± 0.8	0.5 ± 0.8	0.8 ± 1.4	0.545	0.818	0.271
TNF-α (pg/mL)	24.7 ± 7.5	25.4 ± 8.4	28.1± 9.5	0.964	0.913	0.787
IL-6 (pg/mL)	3.2 ± 3.1	3.0 ± 1.8	3.7 ± 3.5	0.629	0.413	0.472
Glucose (mg/dL)	91.3 ± 6.5	91.5 ± 6.7	92.0 ± 6.0	0.271	0.197	0.194
Insulin (μU/mL)	15.1 ± 7.5	14.0 ± 7.9	14.1 ± 6.1	0.740	0.854	0.503
HOMA-IR	3.4 ± 1.8	3.2 ± 1.9	3.2 ± 1.4	0.747	0.983	0.468

Abbreviations: GLM, general linear model; BMI, body mass index; WC, waist circumference; HOMA-IR, homeostasis model assessment of insulin resistance; hsCRP, high-sensitivity C-reactive protein; TNF-α, tumor necrosis factor-α; IL-6, interleukin-6.

Bold indicates significant comparisons (*P* < 0.05).

[a]p1: AA vs. AG vs. GG; p2: AA vs. (AG+GG); p3: (AA+AG) vs. GG, after adjusting for age, Tanner stage, cigarette smoking, alcohol drinking, and physical activity.

Supplemental Table 5. Logistic regression analyses of different *AXL* SNP on abnormal variables among adolescents.

	AXL rs4802113		*AXL* rs2304232	
	TT OR (95% CI)[a]		AA OR (95% CI)[a]	
Variables[b]	Unadjusted	Adjusted[c]	Unadjusted	Adjusted[c]
Boys				
high BMI	0.91 (0.43–1.91)	0.57 (0.32–1.03)	0.81 (0.39–1.66)	0.66 (0.39–1.12)
high WC	1.05 (0.50–2.19)	1.46 (0.58–3.70)	1.32 (0.68–2.58)	1.16(0.46–2.86)
high hsCRP	0.52 (0.24–1.13)	0.59 (0.22–1.56)	1.42 (0.73–2.77)	1.30 (0.54–3.13)
high TNF-α	1.25 (0.55–2.83)	1.27 (0.43–3.85)	1.41 (0.66–3.01)	1.18 (0.40–3.45)
high IL-6	0.74 (0.39–1.41)	0.32 (0.09–1.15)	0.99 (0.55–1.78)	0.93 (0.35–2.44)
high HOMA-IR	0.78 (0.36–1.68)	0.67 (0.23–1.96)	0.76 (0.34–1.55)	0.75 (0.28–1.99)
Girls				
high BMI	0.57 (0.28–1.15)	0.83 (0.43–1.61)	1.35 (0.68–2.66)	1.20 (0.68–2.13)
high WC	0.73 (0.37–1.43)	0.75 (0.29–1.96)	1.27 (0.65–2.48)	0.99 (0.43-2.33)
high hsCRP	0.94 (0.45–1.97)	0.85 (0.34–2.13)	0.85 (0.43–1.66)	0.86 (0.38–1.96)
high TNF-α	1.03 (0.47–2.26)	1.94 (0.75–4.99)	1.05 (0.51–2.16)	1.16 (0.45–2.94)

high IL-6	0.57 (0.26–1.29)	0.51 (0.18–1.41)	0.72 (0.36–1.41)	0.57 (0.24–1.35)
high HOMA-IR	1.23 (0.56–2.72)	1.43 (0.57–3.57)	1.26 (0.60–2.66)	1.26 (0.52–3.03)

[a]Under a recessive model (using heterozygotes and minor homozygotes as the reference for each SNP). [b]Abnormal variables were determined using an age- and gender-specific 90th percentile cut-off point.

[c]Adjusting for age, Tanner stage, cigarette smoking, alcohol drinking, and physical activity.

OR, odds ratio; CI, confidence index; BMI, body mass index; WC, waist circumference; HOMA-IR, homeostasis model assessment of insulin resistance; hsCRP, high-sensitivity C-reactive protein; TNF-α, tumor necrosis factor-α; IL-6, interleukin-6.
